# Outcomes From Health Information Exchange: Systematic Review and Future Research Needs

**DOI:** 10.2196/medinform.5215

**Published:** 2015-12-15

**Authors:** William R Hersh, Annette M Totten, Karen B Eden, Beth Devine, Paul Gorman, Steven Z Kassakian, Susan S Woods, Monica Daeges, Miranda Pappas, Marian S McDonagh

**Affiliations:** ^1^ Pacific Northwest Evidence-Based Practice Center Department of Medical Informatics & Clinical Epidemiology Oregon Health & Science University Portland, OR United States; ^2^ Centers for Comparative and Health System Effectiveness University of Washington Seattle, WA United States; ^3^ Veteran's Affairs Maine Healthcare System Augusta, ME United States

**Keywords:** diagnostic tests, health information exchange, outcome assessment (health care), patient readmission, routine, systematic review

## Abstract

**Background:**

Health information exchange (HIE), the electronic sharing of clinical information across the boundaries of health care organizations, has been promoted to improve the efficiency, cost-effectiveness, quality, and safety of health care delivery.

**Objective:**

To systematically review the available research on HIE outcomes and analyze future research needs.

**Methods:**

Data sources included citations from selected databases from January 1990 to February 2015. We included English-language studies of HIE in clinical or public health settings in any country. Data were extracted using dual review with adjudication of disagreements.

**Results:**

We identified 34 studies on outcomes of HIE. No studies reported on clinical outcomes (eg, mortality and morbidity) or identified harms. Low-quality evidence generally finds that HIE reduces duplicative laboratory and radiology testing, emergency department costs, hospital admissions (less so for readmissions), and improves public health reporting, ambulatory quality of care, and disability claims processing. Most clinicians attributed positive changes in care coordination, communication, and knowledge about patients to HIE.

**Conclusions:**

Although the evidence supports benefits of HIE in reducing the use of specific resources and improving the quality of care, the full impact of HIE on clinical outcomes and potential harms are inadequately studied. Future studies must address comprehensive questions, use more rigorous designs, and employ a standard for describing types of HIE.

**Trial Registration:**

PROSPERO Registry No CRD42014013285; http://www.crd.york.ac.uk/PROSPERO/ display_record.asp?ID=CRD42014013285 (Archived by WebCite at http://www.webcitation.org/6dZhqDM8t).

## Introduction

In recent years, there has been substantial growth in the adoption of the electronic health record (EHR) in ambulatory and hospital settings across the United States, fueled largely by incentive funding provided by the Health Information Technology for Economic and Clinical Health (HITECH) Act. Following HITECH, 94% of nonfederal hospitals [1], 78% of hospital-based physicians [2], 84% of emergency departments (EDs), and 73% of hospital outpatient departments in the United States have adopted EHRs [3]. The motivation to increase the adoption of EHRs is grounded in evidence that health information technology (HIT) can improve the quality, safety, efficiency, and satisfaction with care, as has been reported in a series of systematic reviews [4-7].

One key challenge to effective use of HIT, however, is that most patients in the United States, especially those with multiple conditions, receive care across a number of settings [8,9]. To enable data to follow patients wherever they receive care, attention has recently focused on health information exchange (HIE), defined as the reliable and interoperable electronic sharing of clinical information among physicians, nurses, pharmacists, other health care providers, and patients across the boundaries of health care institutions, health data repositories, laboratories, public health agencies, and other entities that are not within a single organization or among affiliated providers [10].

The Office of the National Coordinator for Health Information Technology (ONC) has defined the following forms of HIE [11]:

1. *Directed exchange:* Sending and receiving secure information electronically between care providers.
2. *Query-based exchange:* Provider-initiated requests for information on a patient from other providers.
3. *Consumer-mediated exchange:* Patients aggregating and controlling the use of their health information among care providers.

ONC also uses the words “push” to describe directed exchange and “pull” to describe query-based exchange [12]. ONC leadership has also advocated that HIE be thought of as a verb and not as a noun, with more focus on the action of exchange and what is achieved with the information than on the technological and organizational structures required [13]. This is not meant to imply that the structures are not necessary, rather it is designed to shift the focus when evaluating HIE from documenting what has been created to the impact HIE has on health and health care.

The HITECH Act recognized that EHR adoption alone was insufficient to realize the full promise of HIT, allocating US $563 million for states or state-designated entities to establish HIE capability among health care providers and hospitals [11]. As a result of HITECH funding, HIE adoption has grown in a parallel though somewhat smaller manner. By 2014, 76% of US hospitals had engaged in some form of HIE [14]. An annual survey of organizations engaged in HIE found 135 in the United States in 2014 [15].

Evaluating the effectiveness of HIE (and HIT generally) has been challenging [16]. HIE systems are intermediate to improving care delivery, allowing clinicians and others improved access to patient data to inform decisions, and facilitate appropriate use of testing and treatment. HIE is not specific to any health issue or diagnosis. HIE implementations have often been supported by one-time start-up funding, without long-term support to sustain the programs long enough for evaluation.

There are 3 previously published systematic reviews that focus exclusively on HIE [17-19]. One of these reviews was conducted a half-decade ago [17], another focused only on US-based and clinical-only (ie, not public health) activities [18], and a third assessed mainly the associations between study characteristics and the frequency of positive outcomes [19]. We expanded upon these reviews to not only perform a systematic review of HIE but also determine needs for future research that reflect our assessment of the benefits and limitations of HIE.

## Methods

Key questions guiding this review were developed by the review team with input from a group of stakeholders and the Agency for Healthcare Research and Quality (AHRQ). A standard protocol was developed using input from key informants and a technical expert panel, registered in PROSPERO [20], and posted on an AHRQ public website. A technical report further describes the methods and includes search strategies and additional information [21]. A research librarian conducted electronic database searches identifying relevant articles published between January 1990 and February 2015 in MEDLINE (Ovid), PsycINFO, CINAHL, and the Cochrane Library databases. Searches were peer reviewed by another librarian and supplemented by references identified from additional sources, including reference lists, table of contents of journals not indexed in databases searched, gray literature sources, and experts. English-language studies of HIE that reported on clinical, economic, population, and intermediate (eg, patient or provider perceptions, availability or accuracy of data, or time saved) outcomes were included. We included comparative studies of effectiveness, and other designs for more qualitative outcomes. We excluded studies that investigated benefits of HIE other than in clinical or public health settings (eg, to enhance clinical research). Two investigators independently evaluated each study to determine inclusion eligibility. Disagreement was resolved by consensus with a third investigator making the final decision as needed.

Details of included studies were extracted by one investigator and reviewed for accuracy and completeness by a second investigator. Two investigators independently assessed risk of bias for all effectiveness studies. Differences were resolved by discussion and consensus and reviewed by the team of investigators. Individual studies were rated as “low,” “moderate,” or “high” risk of bias. Investigators then assessed the strength of the body of evidence. Both the risk of bias and strength of evidence ratings were conducted using the criteria and procedures described in the AHRQ Methods Guide for Effectiveness and Comparative Effectiveness Reviews [22].

The strength of evidence consisted of the following 4 major categories: high, moderate, low, or insufficient, based on the methodological limitations of studies; consistency across studies; precision of estimates; and directness of effect. Ratings were reviewed by a second investigator, and disagreements were resolved by consensus or involvement of a third investigator if necessary. Data could not be combined in a quantitative meta-analysis because of heterogeneity in the interventions, the outcomes measured, and the way data were reported. Therefore, we combined studies qualitatively based on the similarity of the type of HIE, the implementation of the HIE, outcomes measured, and results reported. Where studies were not similar in these areas, we provided the results of the individual studies without grouping them.

## Results

Of the 5211 potentially relevant citations identified in our literature searches, 849 articles were selected for full-text review and 34 studies were ultimately deemed to address outcomes. Study characteristics, results, and risk of bias assessments are presented in Multimedia Appendices 1 and 2. Of the studies included in this report, 2 were randomized controlled trials (RCTs) described in 3 papers and 32 were observational and survey studies. Most were conducted in the United States, although 8 were from Europe, Canada, Israel, and South Korea. These studies reported clinical or public health process, economic, or population outcomes; however, none of the studies explicitly stated that they assessed for harms of HIE or reported any negative unintended consequences. The majority were assessed to be of low risk of bias (ie, good internal validity) but also contained mostly retrospective observational evidence.

Of 34 studies, 26 reported clinical, economic, or population outcomes (see Multimedia Appendix 1), whereas the other 8 were found to report on perceptions of outcomes (see Multimedia Appendix 2). None of the studies evaluated primary clinical outcomes from HIE (eg, mortality and morbidity) nor explicitly measured or reported harms. We list the study designs and geographic locations in Table 1.

The most common study design for assessing outcomes was retrospective cohort, typically with HIE use associated with a specific outcome (Table 1). The next most common design was survey, which was usually focused on perception of effectiveness and perceived outcomes: 2 studies were RCTs—1 RCT assessed a particular directed information exchange (2 published papers, 1 on clinical outcomes, and 1 on perceptions) and the other evaluated a clinical decision support intervention using data from an HIE implementation. Two studies used cross-sectional analyses of large databases to compare health care organizations having access to HIE with those without access. Two other studies used a case series methodology, one of which involved asking clinicians if HIE access avoided undesirable resource use, and then calculating the costs saved and the other that retrospectively analyzed data to determine duplicative testing averted.

The identified studies were performed mostly in the United States, but we identified 8 studies from 5 other countries. Of the 26 studies in the United States, 2 assessed multiple HIE implementations across the entire United States, 1 assessed multiple HIE implementations in 2 states (California and Florida), and the remaining 23 studies were conducted in 13 states. Most studies used retrospective designs, usually with an approach examining the association of HIE use with 1 or more clinical variables. All of these studies focused on the direct effect of HIE, usually reporting reduction in resource use or costs, without determining its larger impact (eg, overall total or proportion of spending in an ED vs the total dollars that HIE appeared to save). None of the studies analyzed individual episodes of care to determine clinical appropriateness of possible changes brought about by HIE use.

The prospective studies also had limitations. The 2 RCTs (reported in 3 papers) were focused on highly specific uses of HIE, namely, directed exchange of ED reports in one and pharmacotherapy clinical decision support in another. Of note, however, was that neither study showed benefit of HIE. The other prospective study was a case series that was limited by its methodology relying on physician self-reports of resources not utilized when HIE was used, with no follow-up or validation of their decisions, or analysis of more holistic views of clinical outcomes or costs.

**Table 1:** Study designs and locations.

	Study designs and locations	References
**Designs (number)**			
	Retrospective cohort (18)	[23-40]
	Survey (8)	[41-48]
	Randomized controlled trial (2 reported in 3 papers)	[49-51]
	Cross sectional (2)	[52,53]
	Case series (2)	[54,55]
**Location (number)**			
	Austria (1)	[47]
	Canada (2)	[49,51]
	Finland (2)	[23,46]
	Israel (2)	[29,56]
	South Korea (1)	[48]
	All of United States (2)	[41,53]
	California and Florida	[52]
	Colorado (1)	[24]
	Indiana (3)	[35,36,44]
	Louisiana (1)	[34]
	Massachusetts (1)	[45]
	Minnesota (1)	[55]
	North Carolina (1)	[50]
	New York (6)	[32,33,37,40,42,43]
	Oklahoma (1)	[38]
	South Carolina (1)	[54]
	Tennessee (3)	[25,27,28]
	Texas (1)	[31]
	Virginia (1)	[39]
	Wisconsin (2)	[26,30]

Most of these studies had reasonable but not strong internal validity. As the intervention (HIE) was only one of many potential influences on clinical outcome (ie, many more factors go into clinical outcomes than the decision to consult an HIE on a patient), there was possible confounding. Because no confounders were explicitly identified and incorporated into the analyses, most studies with appropriate retrospective methods were rated as having low or moderate risk of bias.

Because of the type of study designs used, reporting limitations, and the lack of ability to combine results, the strength of this body of evidence was rated as low, meaning that future studies have the potential to alter these findings in magnitude or direction. In addition, the number of studies and their locations in the United States represent a small fraction of functioning HIE systems. A larger number are reported to be operational, sustainable, or innovating according to the eHealth Initiative Annual Data Exchange Survey, which reported a total of 84 such HIE implementations in 2013 [57] and 106 in 2014 [15]. In other words, while a substantial number of HIE implementations exist in the United States, only a small number have been subject to evaluation. This low number of studies relative to HIE efforts also makes it difficult to generalize about what aspects of HIE, such as location, type, and setting, are associated with the results reported in research.

### Improving Resource Use

Most of the studies of HIE effectiveness focused on resource use. We categorized these as follows (Table 2): laboratory testing, radiology testing, hospital admissions, hospital readmissions, referrals and consultations, ED costs, public heath reporting, quality of care, and other aspects of HIE. Although the risk of bias in most studies was low to moderate, the resulting evidence from them was mostly of low strength due to retrospective designs. This low-strength evidence mostly favored the value of HIE in reducing resource use and costs, especially in the ED, but used a very narrow cost perspective and did not account for how HIE was used and its impact on the overall care of the patient beyond the immediate setting where it was used.

**Table 2:** Study results by categories.

Category (number)	Results
Laboratory testing (6)	A total of 6 studies showed benefit for health information exchange (HIE) in reducing overall testing, although estimates of impact on cost were mixed [23-26,54,55]: 4 studies took place in the emergency department (ED) setting, all showing some amount of reduced testing and cost savings [25,26,54,55], whereas 2 studies were conducted in ambulatory settings, with one showing an increase [23] and the other showing a reduction in the increased overall rate of testing [24].
Radiology testing (9)	A total of 7 studies carried out in the ED setting showing reduced testing [25-28,52,54,55]; 2 studies were conducted in ambulatory settings, with one showing a decrease [23] and the other showing no change in the rate of testing [24].
Hospital admissions (8)	A total of 2 studies found a reduction in hospital admissions and lower costs [25,54]; 3 other studies also measured some benefit for HIE use in reducing hospital admissions [29,32,56], although 3 additional studies found no such reduction [30,31,49].
Hospital readmissions (2)	Whereas 1 study showed benefit for HIE in reducing hospital readmissions [33], the other did not [53].
Referrals and consultations (2)	A total of 2 studies assessed HIE for reducing referrals and/or consultations, with conflicting results [23,54].
ED costs (2)	A total of 2 studies found reduced overall ED costs per patient when HIE was available [25,26]. Neither study reported overall ED expenditures, making it unknown what proportion of overall ED spending was impacted by HIE.
Public heath reporting (3)	A total of 3 studies assessed HIE in public health settings, all of which were conducted in the United States and reported improved automated laboratory reporting [36], improved completeness of reporting for notifiable diseases [35], and improved identification of HIV patients for follow-up care [34].
Quality of care in ambulatory settings (3)	A total of 2 retrospective studies found HIE associated with improved quality of care [37,38], whereas a randomized controlled trial focused on medication reconciliation found increased ability to detect medication adherence problems, the results did not show improvement in adherence after it was identified and addressed by providers [50].
Other aspects of HIE (3)	A total of 3 studies assessed other aspects of HIE, including reduction in time for processing of Social Security Disability claims [39], increased ability to identify frequent ED users [40], and associated HIE implementation with improved patient satisfaction scores in hospitals [41].

### Perceptions

A number of studies evaluated clinician or patient perceptions of outcomes of HIE (see Multimedia Appendix 2), with all reporting perceptions that HIE leads to some benefit including improved outcomes. Clinician perceptions of the value of HIE, where studied, were generally positive. However, how such perceptions translate into improved care is unknown. This body of evidence was considered low strength.

### Factors Associated With Outcomes

To determine whether effectiveness of HIE varied by study type, health care setting, location, or HIE type, we examined whether HIE was found to have some beneficial effect or not across characteristics. As presented in Table 1, the preponderance of studies reported that HIE use for different functions, in various settings, and of varying types produced mostly positive outcomes. Although the number of positive versus negative studies was not an indicator of the overall direction of the evidence, we did note that for each “negative” study, there was at least one “positive” one. For type of HIE, there was no clear pattern of findings to suggest that one type was clearly better than another, even indirectly. The 2 RCTs reported no benefit for their selected outcomes from HIE intervention [49,50], although a perceptions study from one of them reported impressions of improved patient outcomes and management [51]. These were in contrast to the observational study designs where almost all found beneficial effects of HIE. For the HIE setting, only ambulatory and ED had enough studies to evaluate patterns, with outpatient settings less likely to find beneficial results compared with studies in ED settings. The sparseness of studies across geographic settings did not allow for identification of patterns, although across most studies in the United States, the findings were positive.

## Discussion

A collection of low-quality evidence supports the value of HIE for reducing duplicative laboratory and radiology test ordering, lowering ED costs, reducing hospital admissions (less so for readmissions), improving public health reporting, increasing ambulatory quality of care, and improving disability claims processing. The evidence is low quality because of the retrospective nature of the studies and the limited questions that they ask. It is unlikely that additional studies of the kind included in this review will advance the field and strengthen our understanding when HIE can reduce laboratory and imaging tests associated with episodes of care without broadening their scope and using more rigorous designs. Although the preponderance of evidence reports positive effects of HIE in reducing resource use and improving quality of care, it is entirely possible that focused studies with stronger study designs and more comprehensive assessment of utilization or clinical outcomes might reach a different conclusion.

We found no studies explicitly addressing patient-specific clinical outcomes such as morbidity, mortality, or functional status, and therefore the body of evidence is insufficient to determine whether HIE has an impact on patient outcomes. We also did not identify any studies that used systematic and comprehensive economic analysis. Although some of the studies we included projected or estimated cost savings based on measured changes in utilization or perceptions of clinicians, there were no studies that explicitly measured costs and assessed economic impact in a comprehensive fashion. It is fair to say, then, that there was insufficient evidence to reach conclusions on the economic impact of HIE.

### Applicability

How likely are the effects reported in this review to be observed when applied under diverse conditions in health systems, hospitals, and clinics in the United States? The greatest confidence in the applicability of these findings comes from the breadth of settings—geographic, organizational, and technical—from which they are derived. By contrast, there are limitations to the applicability of the findings (beyond limitations to the internal validity already mentioned) having to do with these main concerns: (1) concentration of evidence from a relatively small number of HIE systems; (2) use of internally developed and refined health IT systems compared with local instances of commercial systems; and (3) the exceptionally broad variety of systems, contexts, and purposes of HIE reported in the studies included in this review.

First, the concern that the bulk of the evidence about health IT impact arises out of a relatively small number of centers has been raised before [4]. These centers have been referred to as “health IT leaders,” which are typically large academic medical centers with internally developed health IT systems, implemented incrementally, and refined over a long period. The nature of the health IT systems is in each case unique (being locally developed), and more importantly it is difficult to separate the effects of the health IT from the confounding influences of the health system itself. However, whether findings from these systems can be generalized to the very different context of health system and hospital implementations of commercially developed systems over shorter periods with less internal development and implementation infrastructure has been called into question [4]. This “health IT leader” effect appears to be reduced in more recent updates to the 2006 systematic review by Chaudhry et al [4] but the issue remains important [5,7]. In this review of HIE, the concentration of evidence phenomenon is also present, with large numbers of published studies emanating from relatively few areas, this time regional implementation programs rather than academic health centers, such as Texas, New York, and the MidSouth e-Health Alliance.

Second, separate from the “health IT leader” concern, which has to do with the organizational capacity, resources, and mission of these centers, is the issue of internally developed systems compared with commercially developed systems. Although few of the studies we included described whether their software used was commercial or locally developed, the overall model of health IT purchase and installation of nonhealth IT leaders are usually quite different from that of the incremental internal development, implementation, and refinement that are seen in systems such as the Department of Veterans Affairs or the aforementioned “health IT leader” systems. Related to this concern is a finding from other aspects of health IT [58], namely, clinical decision support, where systems evaluated by their developers tend to achieve more positive outcomes from their evaluation than external evaluators. This phenomenon must be assessed with HIE as well.

Third, and most important, in terms of limiting the applicability of these findings about HIE to real-world use is the exceptionally wide variety of systems, purposes, and contexts of use. To predict whether specific implementations of HIE in specific health care contexts will have favorable impacts on specific desired outcomes is not possible from this review and in most cases would not be possible from comparison with individual studies because (1) it is unlikely that studies with low risk of bias have been published for most such specific questions, and (2) in almost all cases these are complex interventions that are incompletely specified, with insufficient detail to draw strong meaningful inferences [59].

### Limitations of the Evidence Base

The significant limitations of the evidence base, that is, the individual studies included in this review, have been raised in previous systematic reviews of health IT [4,5,7] and of HIE [18]. There are four primary concerns about the limitations of the available evidence on the impact of HIE (and health IT in general): (1) suitability of study design; (2) execution of the studies; (3) complexity of the interventions with implications for interpretation and for generalizability; and (4) changes in the technology or policy governing its use.

First, the evidence in this area addresses a wide variety of questions covering diverse domains beyond medical science from computer science, human factors, sociology, organization and management, and other disciplines. This broad array of questions calls for an equally diverse range of study designs. Studies of usability and use require usability engineering methods, studies of individual behavior call for methods from anthropology and behavioral sciences, studies of organizational change warrant methods drawn from management and systems science, whereas studies of population effects call for the methods of epidemiologists. A significant limitation of this literature, with its breadth of research questions, is the limited toolbox often drawn upon to answer them.

The second limitation is in execution of the studies. Even when strong study designs are used, their execution may be lacking, whether in sampling strategies, measurement methods, or analytic approaches. The unit of analysis problem is but one example. Interventions carried out at the level of the health system, hospital, or clinic may be analyzed at the level of the patient or episode, without controlling for variation at these multiple levels. Incomplete measurement is another: for example, where ED test ordering is measured in isolation, ignoring the possibility that the same test might later be ordered in another setting such as urgent care, primary care, or in hospital.

The third limitation has to do with the complexity of interventions, where the HIE or other health IT system itself is necessarily only part of a more complex intervention. The complexity of interventions to change the behavior of clinicians or others in the health systems studied requires more thorough specification, to both adjust for confounders and make sense out of how to apply interventions elsewhere. Others have documented the inadequacy of specification of the details of complex interventions and called for a more systematic and thorough reporting [59,60].

Finally, the literature does not comprehensively cover changes in technology or policies governing its use. For example, whereas most studies come from the locally developed systems of HIE leaders as noted earlier, there has been a more recent growth in the commercial marketplace for HIE. In addition, the widespread adoption of EHRs under the HITECH Act in the US means that a more diverse array of health care organizations will be participating in HIE implementations. As an example of policy changes governing HIE development, as noted in Table 1, most studies have been of query-based systems whereas more recent meaningful use criteria for incentive funding call for implementation of directed exchange.

### Future Research Needs

Given the limited conclusions that can be reached after review of the large volume of published literature on HIE, what are the implications for future research? Recognizing that HIE, like health IT in general, will almost certainly undergo increasingly widespread implementation in the future, the first aim of researchers should be to shift the emphasis from *whether* HIE systems should be implemented to specifically *how* they should be implemented. The question to be answered is not “Does HIE have positive effects?” but rather “How can HIE be implemented in order to result in the greatest benefit for patients, clinicians, and health systems with the least cost and harm?”

A second aim of research on HIE should be to develop greater focus and clarity about the level at which interventions are operating and the types and levels at which outcomes are measured. The outcomes of interest and the factors influencing them may be quite different at different levels of analysis, from specific systems or functionalities of HIE to individual patients, providers, or episodes of care; to health care units such as the ED, primary care practice, or hospital ward; to institutions such as hospitals; to aggregates such as health systems; or to broader regional multiorganization entities or regions. Combining or confusing these levels of intervention and levels of analysis only increases the challenges for those who conduct the research and for those who wish to interpret and apply it.

To help achieve an improved focus and clarity, a more formal analytic framework and a more descriptive taxonomy are needed. An example of such a framework that could be usefully applied in this area is Rasmussen’s sociotechnical hierarchy, which specifies the multiple levels of a complex sociotechnical system that must be considered together to understand system behavior change [61]. Examples of its application include Vicente’s analysis of the forces acting at multiple levels to reduce hazards arising from patient-controlled analgesia devices [62] and Leveson’s Systems—Theoretic Accident Modeling and Processes model for understanding system performance and safety [63].

Similarly, a formal taxonomy for implementation of complex interventions has been proposed that would enable more complete and useful specification of interventions to allow better analysis, interpretation, and application [59,64]. This taxonomy should be extended specific to HIE to include clinical, technical, and organizational details of the HIE implementation as outlined by Vest [65]. The clinical taxonomy should focus not only on patient outcomes, but also on issues such as health disparities related to HIE and health system issues that may improve or undermine use of HIE. The technical taxonomy should include aspects of system architecture, messaging and terminology standards, and other details. It should also address the financial aspects of implementations, such as whether locally developed or commercial software is used and whether the HIE organization is public or private. The HIE research community should consider a standardized reporting instrument for HIE evaluation comparable to the Consolidated Standards of Reporting Trials statement for RCTs [66].

The third step researchers can take to improve the evidence base for implementation of HIE is to broaden the methodologic toolbox applied to these questions. As indicated earlier, the study approach and architecture must be suited to the question being asked, employing methods from usability engineering, behavioral sciences, systems engineering, and organizational sciences, depending on the question being addressed. These would include methods used in engineering and quality improvement, as well as in the study of complex adaptive systems.

What types of studies should be performed? RCTs are impractical for technologies with wide-ranging purposes like HIE. Yet, retrospective studies associating HIE versus nonuse for outcomes such as test ordering and hospital admissions are very limited in conclusions that can be drawn. Research is also challenging because many of the important clinical outcomes that could be positively affected by HIE have many other potential contributing and confounding factors relating to the patient, his or her clinicians, the quality of care delivered, the EHR, other health IT used, the nature of the health care delivery system, and the regulatory environment. Given the growing evidence based on robust evaluations in other areas of health IT, as noted in systematic reviews [7], methodological insights can be gleaned from other topic areas.

Future studies should be prospective, carried out in mature HIE settings, specify a priori what patients and/or use cases are likely to benefit from HIE, and compare appropriate outcomes for the use or nonuse of HIE. The prospective collection of data from diverse settings where HIE is used, classified by the taxonomy advocated earlier, could allow for prospective cohort studies that could identify aspects of HIE associated with beneficial outcomes. This will likely require an effort comparable in scope to national data collection efforts, such as the Patient-Centered Outcomes Research Institute Clinical Data Research Network initiative [67]. Ideally, such an undertaking could be synergistic with these other large-scale efforts.

Evaluation should be a requirement for all HIE implementations, certainly those funded by grants or other external funding. The challenge of evaluating health IT projects, especially in community settings, is well-known [16], but all funders must demand this requirement to grow the evidence base. By the same token, funders must provide adequate resources for such evaluations. In addition, evaluations should be performed by researchers external to the project to reduce potential bias from system developers evaluating their own implementations [58].

### Conclusions

The full impact of HIE on clinical outcomes and potential harms is insufficiently studied, although evidence provides some support for benefit in reducing use of some specific resources and achieving improvements in quality of care measures. To advance our understanding of HIE, future studies need to address comprehensive questions, use more rigorous designs, and be part of a coordinated, systematic approach to studying HIE. Going forward, HIE will become a more integrated part of health care delivery, and its evaluation needs to be focused on maximizing the improvements that HIE usage brings to overall clinical care.

## Appendix

Multimedia Appendix 1 Studies of health information exchange included for assessing outcomes.

Multimedia Appendix 2 Patient and clinician survey perceptions of health information exchange.

## Abbreviations

AHRQ: Agency for Healthcare Research and Quality
ED: emergency department
EHR: electronic health record
HIE: health information exchange
HIT: health information technology
HITECH: Health Information Technology for Economic and Clinical Health
IT: information technology
ONC: Office of the National Coordinator for Health Information Technology
RCT: randomized controlled trial
