# Supplementary material for: Outcomes From Health Information Exchange: Systematic Review and Future Research Needs
Source: JMIR Med Inform. 2015 Dec 15;3(4):e39. doi: 10.2196/medinform.5215 (PMC4704923; doi:10.2196/medinform.5215)
Supplement: Multimedia Appendix 1 [file medinform_v3i4e39_app1.pdf]

**Multimedia Appendix 1.** Studies of health information exchange included for assessing outcomes.

| | Study | Location | Setting | Health information exchange type | Study type | Risk of bias | Direction of result(s) | Outcome(s) assessed | Results of health information exchange |
|---|---|---|---|---|---|---|---|---|---|
| **Laboratory testing or cost of testing** | | | | | | | | | |
| | Mäenpää et al 2011 [23] | Tampere, Finland | OP[a] | Q[b] | RC[c] | L[d] | Negative | Laboratory testing | Increased testing during period of health information exchange (HIE) implementation (19.0% for primary care physicians and 7.0% for specialist physicians per total patient appointments). |
| | Ross et al 2013 [24] | Mesa County, Colorado | OP[a] | Q[b] | RC[c] | L[d] | Beneficial | Rate of increase in laboratory testing | After HIE implementation, reduction in rising rate of testing, without overall cost savings |
| | Carr et al 2014 [54] | Charleston, South Carolina | Emergency department (ED) | Q[b] | CS[e] | M[f] | Beneficial | Laboratory testing | US $462 in savings over 3 months through averted laboratory testing in EDs |
| | Frisse et al 2012 [25] | Memphis, Tennessee | ED | Q[b] | RC[c] | M[f] | Beneficial | Laboratory testing | Odds ratio (OR) of testing among patients for whom HIE accessed was 0.880 (95% CI 0.828-0.935) |

| | Study | Location | Setting | Health information exchange type | Study type | Risk of bias | Direction of result(s) | Outcome(s) assessed | Results of health information exchange |
|---|---|---|---|---|---|---|---|---|---|
| | Tzeel et al 2011 [26] | Milwaukee, Wisconsin | ED | Q[b] | RC[c] | L[d] | Beneficial | ED visit costs | 23% fewer laboratory testing procedures (statistical significance not reported) in propensity-matched group of patients for whom HIE was used |
| | Winden et al 2014 [55] | Minnesota | ED | Q[b] | CS[e] | M[f] | Beneficial | Laboratory testing | 96 instances of duplicate laboratory testing averted in 1488 patient encounters |
| **Radiology testing** | | | | | | | | | |
| | Bailey et al 2013 [27] | Memphis, Tennessee | ED | Q[b] | RC[c] | L[d] | Beneficial | Use of neuroimaging | HIE usage associated with decreased diagnostic imaging (OR 0.38; 95% CI 0.29-0.50) and increased adherence to evidence-based guidelines (OR 1.33; 95% CI 1.02-1.73), although no significant change in overall costs |

| | Study | Location | Setting | Health information exchange type | Study type | Risk of bias | Direction of result(s) | Outcome(s) assessed | Results of health information exchange |
|---|---|---|---|---|---|---|---|---|---|
| | Bailey et al 2013 [28] | Memphis, Tennessee | ED | Q[b] | RC[c] | L[d] | Beneficial | Use of back imaging | HIE usage associated with reduced repeat imaging for back pain (OR 0.36; 95% CI 0.18-0.71), but no change in cost due to higher use of CT[g] scans with HIE access |
| | Carr et al 2014 [54] | Charleston, South Carolina | ED | Q[b] | CS[e] | M[f] | Beneficial | Use of radiology testing | US $161,000 in savings over 3 months through averted radiologic testing in EDs |
| | Frisse et al 2012 [25] | Memphis, Tennessee | ED | Q[b] | RC[c] | M[f] | Beneficial | Use of radiology testing | Reduction of head CT[g] imaging (OR 0.913; 95% CI 0.842-0.991) and body CT[g] imaging (OR 0.886; 95% CI 0.828-0.948) but no significant changes in echocardiogram, chest X-ray, or ankle X-ray testing across 12 EDs |

| | Study | Location | Setting | Health information exchange type | Study type | Risk of bias | Direction of result(s) | Outcome(s) assessed | Results of health information exchange |
|---|---|---|---|---|---|---|---|---|---|
| | Lammers et al 2014 [52] | California and Florida | ED | Varied | XS[h] | L[d] | Beneficial | Reimaging in ED | Reduced probability of repeat CT[g] (-8.7%; 95% CI -14.7% to -2.7%), ultrasound (-9.1%; 95% CI -17.2% to -1.1%), and chest X-ray (-13.0%; 95% CI -18.3% to -7.7%) ordering in hospitals that had HIE participation |
| | Mäenpää et al 2011 [23] | Tampere, Finland | OP[a] | Q[b] | RC[c] | L[d] | Beneficial | Use of radiology testing | Reduction in radiologic testing: 16.4% reduction for primary care physicians and 11.0% reduction for specialist physicians |
| | Ross et al 2013 [24] | Mesa County, Colorado | OP[a] | Q[b] | RC[c] | L[d] | None | Use of radiology testing | No statistically significant reduction in the rate of radiologic testing |
| | Tzeel et al 2011 [26] | Milwaukee, Wisconsin | ED | Q[b] | RC[c] | L[d] | Beneficial | ED visit costs | 22% decreased diagnostic radiology ordering and 52% reduced CT[g] scan ordering (statistical significance not reported) when HIE was used in ED |

| | Study | Location | Setting | Health information exchange type | Study type | Risk of bias | Direction of result(s) | Outcome(s) assessed | Results of health information exchange |
|---|---|---|---|---|---|---|---|---|---|
| | Winden et al 2014 [55] | Minnesota | ED | Q[b] | CS[e] | M[f] | Beneficial | Use of radiology testing | 453 instances of duplicate radiology testing averted in 1488 patient encounters |
| **Hospital admissions** | | | | | | | | | |
| | Ben-Assuli et al 2013 [29] | Israel | HMO[i] | Q[b] | RC[c] | L[d] | Beneficial | Hospital admissions | Viewing medical history via electronic health record (EHR) decreased possibly redundant admissions, with even greater reductions of 48% at 7 days when information was accessed using HIE |
| | Ben-Assuli et al 2013 [56] | Israel | HMO[i] | Q[b] | RC[c] | L[d] | Beneficial | Hospital admissions | Viewing medical history via EHR decreased possibly redundant admissions, with even greater reductions of 6.0% at 7 days when information was accessed using HIE |
| | Frisse et al 2012 [25] | Memphis, Tennessee | ED | Q[b] | RC[c] | L[d] | Beneficial | Hospital admissions | US $1.07 million annual savings, with 97.6% due to reduced admissions |

| | Study | Location | Setting | Health information exchange type | Study type | Risk of bias | Direction of result(s) | Outcome(s) assessed | Results of health information exchange |
|---|---|---|---|---|---|---|---|---|---|
| | Carr et al 2014 [54] | Charleston South Carolina | ED | Q[b] | CS[e] | M[f] | Beneficial | Hospital admissions | US $118,000 in savings from averted admissions over a 3-month period |
| | Tzeel et al 2012 [30] | Milwaukee, Wisconsin | ED | Q[b] | RC[c] | L[d] | Mixed | Hospital admissions<br>Length of stay | 28% increased rate of admissions, although such admissions had reduced length of stay with 771 fewer bed days per 1000 health plan members over 16 months |
| | Vest 2009 [31] | Austin, Texas | ED | Q[b] | RC[c] | L[d] | Beneficial | Hospital admissions for ambulatory-sensitive diagnoses in indigent patients | Increased admissions for ambulatory-sensitive diagnoses |
| | Vest et al 2014 [32] | Rochester, New York | ED | Q[b] | RC[c] | L[d] | Beneficial | Hospital admissions | Viewing information reduced odds of admission (OR 0.70; 95% CI 0.52-0.95). |
| **Hospital/ED readmissions** | | | | | | | | | |

| | Study | Location | Setting | Health information exchange type | Study type | Risk of bias | Direction of result(s) | Outcome(s) assessed | Results of health information exchange |
|---|---|---|---|---|---|---|---|---|---|
| | Lang et al 2006 [49] | Montreal, Canada | ED | D[j] | Randomized controlled trial (RCT) | M[f] | None | ED return visits | Providing family physicians electronic reports of ED visits versus paper-based reports resulted in no difference in hospital admissions or ED return visits |
| | Vest et al 2014 [33] | Rochester, New York | ED | Q[b] | RC[c] | L[d] | Beneficial | Hospital readmissions | Accessing information associated with reduced odds of hospital readmission (OR 0.43; 95% CI 0.27-0.70) |
| | Jones et al 2011 [53] | United States | All | Varied | XS[h] | L[d] | None | Hospital readmissions | US hospitals participating in HIE in 2007 did not have lower readmission rates for acute myocardial infarction, pneumonia, or heart failure |
| **Referrals and/or consultations** | | | | | | | | | |

| | Study | Location | Setting | Health information exchange type | Study type | Risk of bias | Direction of result(s) | Outcome(s) assessed | Results of health information exchange |
|---|---|---|---|---|---|---|---|---|---|
| | Carr et al 2014 [54] | Charleston, South Carolina | ED | Q[b] | CS[e] | M[f] | Beneficial | Consultation | US $4000 in savings over 3 months through averted consultations in EDs |
| | Mäenpää et al 2011 [23] | Tampere, Finland | OP[a] | Q[b] | RC[c] | L[d] | Mixed | Referral ordering | Increased referrals by primary care physicians (43.6%) and specialists (12.8%) |
| **ED costs** | | | | | | | | | |
| | Frisse et al 2012 [25] | Memphis, Tennessee | ED | Q[b] | RC[c] | L[d] | Beneficial | Overall cost | US $1.07 million annual savings, with 97.6% due to reduced admissions |
| | Tzeel et al 2011 [26] | Milwaukee, Wisconsin | ED | Q[b] | RC[c] | L[d] | Beneficial | ED visit costs | US $29 per ED visit less expenditures, driven by reduced laboratory testing |
| **Public health reporting** | | | | | | | | | |
| | Magnus et al 2012 [34] | Louisiana | PH[k] | D[j] | RC[c] | L[d] | Beneficial | Follow-up care for HIV patients | Increased identification of needed follow-up care of 419 HIV patients, with 85% having actual follow-up care |

| | Study | Location | Setting | Health information exchange type | Study type | Risk of bias | Direction of result(s) | Outcome(s) assessed | Results of health information exchange |
|---|---|---|---|---|---|---|---|---|---|
| | Dixon et al 2011 [35] | Indiana | PH[k] | D[j] | RC[c] | L[d] | None | Completeness of public health reporting | Equal or improved completeness of reporting for a variety of data fields in notifiable disease reports, although completeness was reduced for some fields (eg, laboratory units of measure, normal range, and abnormal flag) due to inadequacies in the clinical data entering the HIE |
| | Overhage et al 2008 [36] | Indiana | PH[k] | D[j] | RC[c] | L[d] | Beneficial | Identification and completeness of notifiable disease reporting | Automated laboratory reporting through HIE led to 4.4-fold higher rate of reporting, with cases identified an average of 7.9 days earlier compared with usual (spontaneous) public health reporting |
| **Quality of ambulatory care** | | | | | | | | | |

| | Study | Location | Setting | Health information exchange type | Study type | Risk of bias | Direction of result(s) | Outcome(s) assessed | Results of health information exchange |
|---|---|---|---|---|---|---|---|---|---|
| | Kern et al 2012 [37] | Hudson Valley, New York | OP[a] | Q[b] | RC[c] | L[d] | Beneficial | Clinical quality measures | For a benchmark group of clinical quality measures believed to be amenable to HIE, users of HIE had a higher proportion exceeding mean clinical quality measure performance at baseline (57% vs 48%) that increased further after HIE became available (64% vs 49%), with increase before and after availability of HIE statistically significant (*P*<.001) |

| | Study | Location | Setting | Health information exchange type | Study type | Risk of bias | Direction of result(s) | Outcome(s) assessed | Results of health information exchange |
|---|---|---|---|---|---|---|---|---|---|
| | Nagykaldi et al 2014 [38] | Norman and Oklahoma City, Oklahoma | OP[a] | Q[b] | RC[c] | M[f] | Beneficial | Clinical quality measures | Improved documentation and delivery of preventive services for mammography screening (21.1-57.1%, *P*<.01), colonoscopy screening (31.7-53.8%, *P*<.01), pneumococcal vaccine administration (39.1-50.6%, *P*<.01), and influenza vaccine administration (22.7-41.7%, *P*<.01). Also found that medication reconciliation completion improved from 35.3% to 44.9% (*P*<.001). |

| | Study | Location | Setting | Health information exchange type | Study type | Risk of bias | Direction of result(s) | Outcome(s) assessed | Results of health information exchange |
|---|---|---|---|---|---|---|---|---|---|
| | Willis et al 2013 [50] | North Carolina | OP[a] | Q[b] | RCT | M[f] | Beneficial | Documentation and medication reconciliation | HIE data used in a clinical decision support intervention able to detect medication adherence problems in 8 categories of drugs but did not show any benefit in improving adherence by patients in taking medications prescribed based on evidence-based guidelines |
| **Other aspects of HIE** | | | | | | | | | |
| | Feldman and Horan 2011 [39] | Virginia | G[l] | D[j] | RC[c] | M[f] | Beneficial | Case processing time for SSD[m] determination | 30% reduction in evaluation time for Social Security Disability claims |
| | Shapiro et al 2013 [40] | New York | ED | Q[b] | RC[c] | M[f] | Beneficial | Identification of frequent ED users | 20.3% increase in identifying frequent ED users compared with site-specific data |

| | Study | Location | Setting | Health information exchange type | Study type | Risk of bias | Direction of result(s) | Outcome(s) assessed | Results of health information exchange |
|---|---|---|---|---|---|---|---|---|---|
| | Vest and Miller 2011 [41] | US | H[n] | Varied | XS[h] | L[d] | Beneficial | Patient satisfaction with hospital care | Communication and satisfaction (based on the Hospital Consumer Assessment of Healthcare Providers and Systems survey) higher in hospitals that implemented HIE compared with those that proposed to implement HIE |

[a]OP: outpatient

[b]Q: query

[c]RC: retrospective cohort

[d]L: low

[e]CS: case series

[f]M: moderate

[g]CT: computing tomography

[h]XS: cross sectional

[i]HMO: health maintenance organization

[j]D: directed

[k]PH: public health

[l]G: government

[m]SSD: Social Security Disability

[n]H: hospital
