# Supplementary material for: Outcomes From Health Information Exchange: Systematic Review and Future Research Needs
Source: JMIR Med Inform. 2015 Dec 15;3(4):e39. doi: 10.2196/medinform.5215 (PMC4704923; doi:10.2196/medinform.5215)
Supplement: Multimedia Appendix 2 [file medinform_v3i4e39_app2.pdf]

Multimedia Appendix 2. Patient and clinician survey perceptions of health information exchange.

**Study**	**Location**	**Setting**	**Health information exchange type**	**Study type**	**Risk of bias**	**Direction of result(s)**	**Perception(s) assessed**	**Results**
Afilalo et al 2007 [51]	Montreal, Canada	Emergency department (ED)	D[a]	Randomized controlled trial	M[b]	Beneficial	Outcomes improved, better patient management	Family physicians reported improved patient management and follow-up with exchanged summary of ED visit.
Altman et al 2012 [42]	New York	ED	D[a]	XS[c]	M[b]	Beneficial	Primary care provider notification of ED admission/discharge	Primary care physicians reported enhanced awareness and improved communication and follow-up after ED admission/discharge.
Campion et al 2012 [43]	Rochester and Buffalo, New York	OP[d]	Both	XS[c]	M[b]	Beneficial	Physician satisfaction of push vs pull	Physicians reported satisfaction with both push and pull health information exchange, with higher satisfaction for push.
Chang et al 2010 [44]	Indiana	OP[d]	Q[e]	XS[c]	M[b]	Beneficial	Physician satisfaction with electronic laboratory reports	Physicians more satisfied with electronic laboratory reports than paper-based reports.
Kaushal et al 2010 [45]	Massachusetts	ED	D[a]	XS[c]	H[f]	Mixed	Impact of providing pharmacy information	Providing pharmacy information to physicians in the ED improved knowledge and gaps but was not felt to reduce time or be worth the cost.

Study	Location	Setting	Health information exchange type	Study type	Risk of bias	Direction of result(s)	Perception(s) assessed	Results
Maass et al 2008 [46]	Finland	OP[d]	Q[e]	XS[c]	H[f]	Beneficial	Improvements in care	Perceived improvement in ambulatory care function, resulting in faster acquisition and treatment decisions.
Machan et al 2006 [47]	Tyrol, Austria	OP[d]	D[a]	XS[c]	L[g]	Beneficial	Physician satisfaction with discharge reports sent	Improved care and decreased work for filing and archiving discharge reports with health information exchange.
Park et al 2013 [48]	South Korea	OP[d]	D[a]	XS[c]	L[g]	Beneficial	Patient perceptions of data transferred	Improved patient satisfaction when records were transferred via health information exchange over patients delivering paper records themselves.

[a]D: directed

[b]M: moderate

[c]XS: cross sectional

[d]OP: outpatient

[e]Q: query

[f]H: high

[g]L: low
